# Novel Poly(Adenosine Diphosphate-Ribose) Polymerase (PARP) Inhibitor, AZD2461, Down-Regulates *VEGF* and Induces Apoptosis in Prostate Cancer Cells

**DOI:** 10.29252/.23.5.312

**Published:** 2019-09

**Authors:** Saman Sargazi, Ramin Saravani, Javad Zavar Reza, Hossein Zarei Jaliani, Hamidreza Galavi, Mahdiyeh Moudi, Najmeh Alsadat Abtahi

**Affiliations:** 1 International Campus, Shahid Sadoughi University of Medical Sciences, Yazd, Iran;; 2 Biotechnology Research Center, International Campus, Shahid Sadoughi University of Medical Science, Yazd, Iran;; 3 Department of Clinical Biochemistry, School of Medicine, Zahedan University of Medical Sciences, Zahedan, Iran;; 4 Cellular and Molecular Research Center of Zahedan University of Medical Sciences, Zahedan, Iran;; 5 Department of Clinical Biochemistry, School of Medicine, Shahid Sadoughi University of Medical Sciences, Yazd, Iran;; 6 Protein Engineering Laboratory, Department of Medical Genetics, School of Medicine, Shahid Sadoughi University of Medical Sciences, Yazd, Iran;; 7 Clinical Immunology Research Center of Zahedan University of Medical Sciences, Zahedan, Iran;; 8 Genetics of Non-Communicable Disease Research Center of Zahedan University of Medical Sciences, Zahedan, Iran

**Keywords:** AZD2461, DNA repair, PARP, Prostate neoplasm

## Abstract

**Background:**

Prostate cancer (Pca) is a heterogeneous disease, and current treatments are not based on molecular stratification. Poly(adenosine diphosphate [ADP]-ribose) polymerase (PARP) inhibitors have recently been found to be remarkably toxic to cells with defects in homologous recombination, particularly cells with BRCA-mutated backgrounds. Therefore, this preliminary study was designed to evaluate whether PTEN expression status could have an impact on the sensitivity of invasive Pca cells to the PARP inhibitor, AZD2461.

**Methods:**

MTT viability test, Annexin V‐FITC/propidium iodide double staining, and caspase3 activity assay were used to evaluate the apoptosis and relative expression of PTEN and VEGF in PC-3 and DU145 cell lines using real-time PCR.

**Results:**

MTT results showed that the inhibitory effects of AZD2461 were higher in PC-3 than DU145 cells (with IC_50_ of 36.48 and 59.03 µM at 48 hours of treatment, respectively). Flow cytometric analysis also showed the same results. When exposed to 40 µM of AZD2461, PC-3 (38.8%) and DU145 (28%) cells underwent apoptosis (*p* < 0.05). Treatment of cells by AZD2461 also caused a significant increase in apoptosis through caspase3 activation in both cell lines. VEGF mRNA levels in PC-3 cells significantly decreased compared to adjusted untreated cells (*p* < 0.05) in all measured times while displaying different alteration patterns in DU145 cells (*p* < 0.05).

**Conclusion:**

AZD2461 suppresses the growth of prostate tumor cells since AZD2461 monotherapy could prove to be efficacious, especially against cells not expressing PTEN besides activating the possible apoptosis-independent cell death pathways.

## INTRODUCTION

Prostate cancer (Pca), as the most prevalent male malignancy, is responsible for 27,000 deaths in the United States^[^^1^^,^^2^^]^ and over 250,000 deaths worldwide per year. Although the high incidence of Pca is thought to be primarily related to numerous genetic factors, familial history, and age, the main mechanism implicated in driving this fatal disease is not completely found yet^[^^3^^]^. Prostate tumors frequently display incredible clinical heterogeneity as some patients dying of Pca within 24 to 48 months of diagnosis, while others can survive for many years with the organ-confined disease, probably due to genetic diversities. 

Several repeated DNA alterations have been discovered due to Pca genetic profiling. These alterations basically dysregulate genes that are involved in Pca development, chromatin modification, and regulation of cell-cycle alongside other underlying mechanisms^[^^4^^]^. Somatic point mutations including deletions are less common in Pca than other solid malignancies and commonly accumulate in the early stages of Pca carcinogenesis, which normally causes *TP53*, *PTEN*, and *CDKN1B *loss of expression^[^^5^^]^.

Many novel targeted therapies are based on inducing caspase-dependent or -independent cell apoptosis in cancer cells or sensitizing these cells to certain cytotoxic agents^[^^6^^,^^7^^]^. In spite of the fact that germline Breast Cancer Associated (*BRCA*) mutations are quite uncommon in Pca (0.44% for *BRCA1* and 1-2% for *BRCA2*), there are other epigenetic alterations affecting genes involved in DNA repair mechanisms, particularly homologous recombination repair (HRR) pathway^[^^8^^]^. It has also been revealed that alteration frequency of *PTEN* gene is approximately 40% in metastatic prostate samples^[^^9^^]^. Also, Monoallelic loss of *PTEN* is present in 60% of localized Pca cases since the complete loss of *PTEN* in Pca has been linked to tumor invasion^[^^10^^]^.

Advanced Pca often reflects a genomic instability that contributes to DNA damage response defects, which may eventually cause cell death^[^^11^^]^. In contradiction, Pca cells are dependent on certain DNA repair pathways to prohibit DNA damage accumulation in proliferating cells. The most lethal form of DNA damage, double-strand breaks (DSB), are effectively repaired by either non-homologous end joining (NHEJ) or HRR pathway mediated by (BRCA) proteins (specifically BRCA1 and BRCA2) and RAD51 as well^[^^12^^]^. The existence of deletion mutations in HR related genes including PTEN can result in hampered DNA damage response (DDR) of cancer cells, causing these cells to be thoroughly sensitive to DNA perturbations^[^^13^^]^. DDR inhibitors as single agents have been proved to have anti-cancer effects in specific tumor genetic backgrounds based on the principle of synthetic lethality^[^^14^^]^. This approach was primarily employed by using poly(adenosine diphosphate [ADP]-ribose) polymerase (PARP) inhibitors as a DDR protein in BRCA-deficient breast and ovarian cancers^[^^15^^]^. Recent attempts to develop PARP inhibitors, as anticancer drugs, constitute the culmination of over 40 years of research. Amongst 17 members of PARP superfamily, both PARP1 and PARP2 are required to repair DNA single-strand breaks (SSBs) and PARP1 repairs replication fork damages during DNA DSBs^[^^16^^]^. Although PARP1 and PARP2 possess overlapping roles, but their substrate preference is not the same and the cytotoxicity of PARP inhibitors mediates rather by only inhibiting the function of PARP1. Olaparib (AZD2281) is the first potent PARP inhibitor approved by Food and Drug Administration (FDA) as an anti-tumor and anti-invasion agent in patients with BRCA-mutant ovarian, lung and breast cancers^[^^17^^-^^19^^]^. A next-generation PARP inhibitor, AZD2461, a novel small molecule structurally analogous to olaparib, preserves the same level of anti-cancer effectiveness with less sensitivity to drug resistance mechanisms^[^^20^^]^. Studies have suggested that the clinical use of PARP inhibitors might be extended to malignancies other than *BRCA1/2*-associated cancers^[^^21^^,^^22^^]^, highlighting the need for evaluating the response of cells with different expression levels of *PTEN* to PARP inhibition.

It has been reported that PARP inhibitors have anti-angiogenic effects^[^^23^^]^. Accordingly, other possible targets for these inhibitors are growth factors such as VEGF, FGF2, TGFB1, EGF, and IGF1, which are involved in the development of Pca cells and can be potential targets for cancer therapy^[^^24^^]^. Among Pca cell lines, PC-3 cells express much lower levels of *PTEN* and have higher metastatic potential compared to DU145 and LNCaP cells^[^^25^^]^. PC-3 and DU145 cells do not respond to androgens, but studies have indicated that the cells are influenced mostly by epidermal growth factors^[^^26^^]^. In contrast, DU145 is another hormone-insensitive Pca cell line that have significantly higher baseline *PTEN* expression and does not express prostate-specific antigen^[^^27^^]^.

To our knowledge, there is no report on the effects of AZD2461 on apoptosis induction and possible alterations in VEGF mRNA in PC-3 prostate carcinoma cell line expressing very low levels of *PTEN *(due to homozygous mutation) compared to high *PTEN*-expressing DU145 cells. Therefore, in this preliminary experiment, we have studied the effects of the PARP1 inhibitor on the *VEGF* gene expression profile as an indicator of tumor invasiveness in Pca cells. Our results can provide a rationale for the utilization of AZD2461 as a refreshing cytotoxic agent against sex-related solid tumors such as sporadic prostate carcinoma. 

## MATERIALS AND METHODS

**Drugs and chemicals**

AZD2461 and 3-(4,5-dimethylhiazol-2-yl)-2,5-diphenyltetrazolium bromide (MTT) were obtained from Sigma-Aldrich (St. Louis, MO, USA), prepared as stock solutions in PBS and stored at -20 °C until further use. All other reagents and biochemicals, including *antibiotic-antimycotic solution* (penicillin/ streptomycin and amphotericin B), HPLC grade DMSO, Trypan blue, RPMI-1640 medium, and Trypsin were of analytical grade and purchased from Innovative Biotech Co. (INOCLON, Iran). Fetal bovine serum (FBS) was procured from Gibco (Rockville, MD, USA). Annexin V-FITC Apoptosis Detection Kit I was obtained from BD Biosciences (San Jose, CA, USA). cDNA synthesis kit, SYBR Green master mix, and caspase3 colorimetric assay kit were purchased from TaKaRa Biotechnology (Japan), Ampliqon A/S (Odense M, Denmark), and R&D Systems Co. (Grodig, Germany), respectively.

**Cell lines, culture methods, and cell treatment**

The human PC-3 prostate carcinoma cell line was obtained from Pasteur Institute of Iran (IPI), Tehran, Iran and DU145 cells from Cell Repository of the Research Institute of Biotechnology, Ferdowsi University of Mashhad, Mashhad, Iran. Cell lines were cultivated in RPMI-1640 medium containing 10% FBS, streptomycin (100 U/ml), penicillin (105 mg/ml), and amphotericin B (2.5 mg/L). The cells were then maintained under the standard cell conditions (95% humidified air and 5% CO_2_ at 37 °C)^[^^28^^]^ until reaching 80% of confluency. All measurements were done in triplicates.

**Cell proliferation assay**

The cytotoxic effect of AZD2461 inhibitor on the proliferation of PC-3 and DU145 cell lines was determined using MTT assay^[^^29^^]^ to measure the half-maximal inhibitory concentration (IC50) of the inhibitor on both cell lines. The cells were seeded in a 96-well plate at the density of 5500 cells/well. After an overnight incubation, the medium was aspirated, and the cells were treated with AZD2461 at increasing concentrations ranging from 5 µM to 160 µM. Following 24, 48, and 72 h of sample exposure period, 20 μL of tetrazolium dye (5 mg/ml) was added to the control and to all the treated wells and incubated for 2 h; the mixture remained in a humidified atmosphere at 37 °C. After discarding the culture medium, 150 μL of DMSO was added, and the absorbance at 570 nm was measured using a microplate reader (Stat Fax 2100; Awareness Technology, Los Angeles, CA, USA). The cells exposed to 1% DMSO contained culture medium were considered as controls. Results were expressed as the percentage of absorbance in the treated cells divided by the percentage of absorbance in the control cells (vehicle was set at 100%).

**Apoptosis analysis**

Evaluation of apoptosis was performed by the Annexin Vpropidium iodide (PI) double staining assay according to the manufacturer's instructions. Partec PAS-II flowcytometry was used for the analysis of the samples. The extent of apoptosis was expressed as a percentage of Annexin V^+^/PI^- ^(early apoptosis) and of Annexin V^+^/PI^+ ^(late apoptosis)^[^^30^^]^. Twenty four hour after cell seeding (8-10 10^4 ^cells per well), PC-3 and DU145 cells were treated with the concentrations of 10, 20, and 40 μM of AZD2461, separately. After, 48 h of treatment, the cells were washed twice with cold PBS (0.137 mM of NaCl, 2.7 mM of KCl, 8.0 mM of Na_2_HPO_4_, and 1.47 mM of KH_2_PO_4_). The cell pellets were then washed and resuspended in the binding buffer. Afterward, 5 μL of Annexin V-FITC was added to the cell suspension and incubated in the dark at room temperature for 15 minutes. Then 5 μL of PI was added to the suspension in the dark and incubated for 10 minutes. Following gentle pipetting, 400 μL of buffered isotonic solution was added to each sample before analyzing by a fluorescence-activated cell sorting (FACS) cytometer. 

**Caspase3 activity assay**

In order to determine caspase3 activity, colorimetric assay kit was utilized according to the manufacturer’s protocol. Cells (8 10^4 ^per well) were seeded in a 6-well plate. Following 24 h of seeding, the cells were treated with the increasing concentration of AZD2461 from 10 to 40 μM at different times, including 3, 6, 12, and 24 h post treatment. By dividing the absorbance of treated cells by that of control cells, the caspase3 activity was measured and reported as fold change in a concentration- and time-dependent manner^[^^31^^]^.

**Gene expression assay**

**Primer design, total RNA extraction, and cDNA synthesis**

*VEGF* gene sequence was blasted against various NCBI-accessible databases. The designed forward and reverse primer sequences for the analysis of *VEGF* gene expression were 5'-GAAGGAGGAGG GCAGAATCATCAC-3' and 5'-CACAGGATGGCT TGAAGA TGTACTC-3', respectively, as well as for *PTEN *were 5'-CGGCAGCATCAAATGTTTCAG-3' and 5'-AAC TGGCAGGTAGAAGGCAACTC-3'and glyceraldehyde -3-phosphate dehydrogenase (GAPDH; as a reference gene) were 5'-GAGCCATCGTCAG ACAC-3' and 5'-CATGTAGTTGGAATGAAGG-3'), as forward and reverse primers,respectively. The *PTEN* and *VEGF* primers were designed to ensure the amplification of all the isoforms (transcript variants 1 and 2 of *PTEN* and 1 to 10 of *VEGF*). A standard curve was constructed using 5-fold dilution series.

To obtain total RNA, 1 mL RNX (SinaClon, Tehran, Iran) was added to each cell-contained microtube, and total RNA was extracted in 0, 6, 12, and 24 h after treatment. The precipitated RNA was resuspended in 30 μL of diethyl phosphorocyanidate water and incubated at -20 °C overnight before cDNA synthesis. UV absorbance ratios, A^280^/A^260^ and A^230^/A^260^, were used to evaluate DNA content and the purity of the extracted RNA.

Following instruction for cDNA synthesis, a mixture of reagents, including template RNA (~4 μg) from different time points (0 [control], 6, 12, and 24 hours), 0.5 μL of Oligo(dT) primer, 0.5 μl of random 6-mers, and 2.5 μL of RNase-free dH_2_O was prepared in a microtube. After incubation at 65 °C for 5 minutes and immediate cooling on ice, 0.5 μL of PrimeScript^TM^ RT enzyme Mix I and 2 μl of 5 PrimeScript^TM^ buffer were added to a total volume of 10 μL. PCR amplification was performed at 37 °C for 15 minutes, followed by 85 °C for 5 seconds and 10 minutes of holding at 4 °C. cDNA samples were stored at -20 °C until use.

**Real-time PCR analysis**

 Real-Time PCR (ABI Sequence Detection System; Applied Biosystems, Foster City, CA, USA) was used to evaluate *PTEN* and *VEGF* mRNA gene expression pattern in PC-3 and DU145 cells according to the manufacturer's protocol. The *denaturation* temperature was *95 °C* for 10 minutes, followed by 35 cycles: 95 °C for 30 s and 58 °C for 30 s, and the *extension* was carried out at 72 °C for 45 s. The comparative 2^-^^ΔΔCt^ method was employed to quantify *differences* in the expression *level of* *PTEN* and *VEGF*. A non-template control was included in all batches, and all assays were repeated at least in triplicate.

**Statistical analysis**

Data were presented as the mean ± standard deviation (SD) and analyzed by one-way analysis of variance (ANOVA), followed by post-hoc Tukey’s test using the SPSS16 software (SPSS Inc., Chicago, Illinois, USA). IC_50_ values were calculated using Prism software version 6.0 (GraphPad Software Inc., San Diego, CA, USA)^[32]^ from individual experiments.* p* < 0.05 was considered statistically significant.

## RESULTS

**Analysis of cell morphology**

**Fig. 1:**
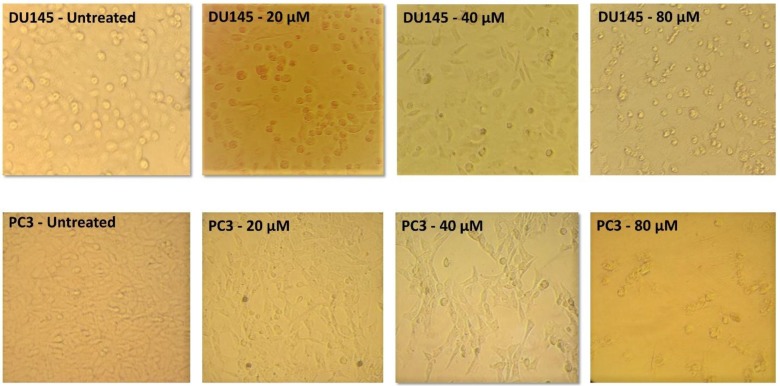
Morphological alterations of DU145 and PC-3. Cells were monitored for 48 h at three different concentrations of AZD2461. Both cells influenced the concentration-dependent cytotoxic effects of AZD2461, but PC-3 cells experienced more evident morphological changes and higher reduction in viability compared to DU145 cells

Following 48 h of treatment with three different concentrations (20, 40, and 80 µM) of AZD2461, the morphology and the number of viable cells of both DU145 and PC-3 cell lines altered in a concentration-dependent manner compared to untreated cells. When treated with 20 µM of AZD2461, the cells were deformed, and with increasing the concentration, dropsy and shrinkage were augmented. In addition, after exposing the cells to 40 µM of AZD2461, approximately half of the cells lost their viability, and by treating both cell lines with 80 µM of this specific inhibitor, the rupture of cell membranes and the release of cytosolic content was distinctly observed. The PC-3 cell line almost experienced the same condition, but the extent of morphological changes and reduction of viable cells were more evident while treating with the same concentrations of AZD2461 (Fig. 1).

***AZD2461 dose-response*** **effect**

**Fig. 2:**
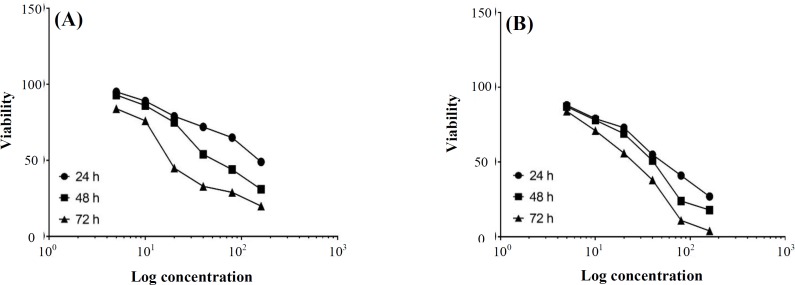
Concentration-response analysis of AZD2461 toxicity after 24, 48, and 72 h post treatment against (A) DU145 and (B) PC-3

**Fig. 3:**
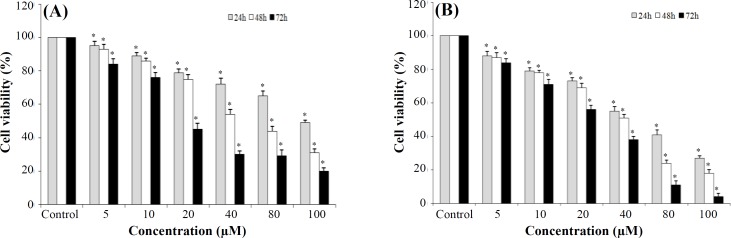
Cytotoxic effects of AZD2461after 24, 48, and 72 h post treatment analyzed by MTT assay on (A) DU145 and (B) PC-3 cell lines.^*^p < 0.05 significance compared to adjacent untreated control. All assays were done in triplicate. The data shows that in same concentrations, PC-3 cells are more sensitive to PARP inhibition by AZD2461 than DU145 cells, which express high levels of *PTEN*

Effects of specific PARP1 inhibitor (AZD2461) on the proliferation of Pca cell lines were evaluated by MTT assay similar to the *dose-response curves for* *DU145 and PC-3 cells (*Fig. 2A and 2B*,* *respectively)*. AZD2461 significantly suppressed the proliferation of both DU145 (Fig. 3A) and PC-3 (Fig. 3B) cells in a time- and concentration-dependent manner (*p *< 0.05). IC50 values calculated by GraphPad Prism software for 24, 48, and 72 h treatment of PC-3 cells with AZD2461 were 51.71, 36.48, and 21.73 µM, while these values were 128.1, 59.03, and 23.69 µM for DU145 cells. The most significant inhibitory effects of AZD2461 on PC-3 cells were 55.2 ± 2.69%, 51.0 ± 2.1%, and 56.1 ± 2.66% after treatment for 24 h (at 40 µM), 48 h (at 40 µM), and 72 h (at 20 µM), respectively. Regarding the effects of AZD2461 on DU145 cells, treatment with AZD2461 caused the reduction of 49.1 ± 1.38%, 54.2 ± 2.98%, and 44.9 ± 3.63% in cell viability after 24 h (at 160 µM), 48 h (at 40 µM), and 72 h (at 20 µM), respectively (Fig. 3). 

**Induction of apoptosis in Pca cell lines by AZD2461**

**Fig. 4:**
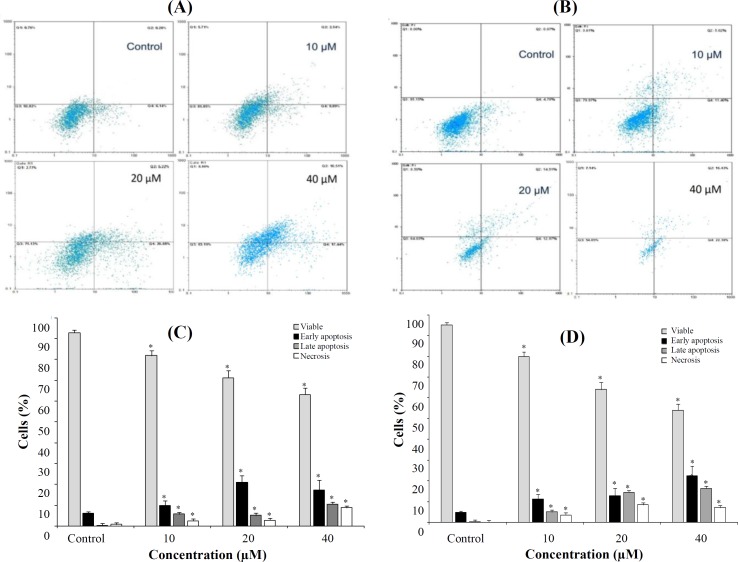
Flow cytometric evaluation of apoptosis in DU145 (A and C) versus PC-3 (B and D) cells using Annexin V‐FITC/PI V and propidium iodide double staining. After 48 hours of treatment, PARP inhibition caused by AZD2461 resulted in a substantial increase in both the early and late apoptotic phase of two cell lines, in a concentration-dependent manner. Treatment by 10, 20, and 40 µM of AZD2461 caused marked apoptosis in both Pca cell lines, but the apoptotic rate was higher in PC-3 cells while raising the possibility of necrosis induction and treating both cells with higher concentrations of AZD2461. The data were expressed as mean ± SD, three independent experiments. Error bars present standard deviation. ^*^p < 0.05 compared with the control

AnnexinV-PI staining was used to identify apoptotic cells following 48 h of treatment (Fig. 4). Although results acquired by this method were similar to those obtained with the MTT assay, the type of cell death was not very different among two cell lines. Compared to the control values (6.14% for DU145 and 4.78% for PC-3 cells), the percentages of early apoptotic cells (AnnVpos/PIneg) were 9.89%, 20.88%, and 17.44 % for DU145 and 11.40%, 12.97%, and 22.38% for PC-3 cells, when treated with 10, 20, and 40 µM of AZD2461, respectively. As shown in Figure 4C and 4D, AZD2461 significantly increased the number of late apoptotic cells (AnnVpos/PIpos) and total apoptotic cells in both cell lines in a concentration-dependent manner, resulting in 27.95% and 38.81% total apoptotic DU145 and PC-3 cells, when both cell lines were treated with 40 µM of AZD2461 (*p *< 0.05). *The percentage of cells underwent necrosis was also found to be increased in DU145 and PC-3 cells, but not in a concentration dependent manner.*

**Caspase3 activity assay**

**Fig. 5:**
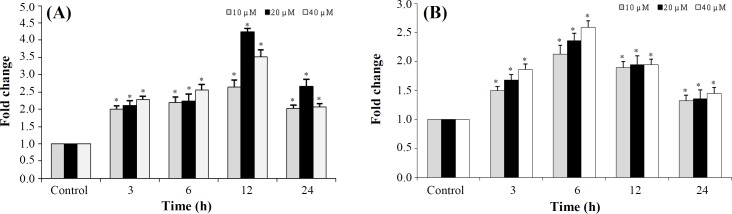
Effect of AZD2461 on caspase-3 activity in A) PC-3 and (B) DU145cells. Cells were incubated at a concentration of 10, 20, and 40 µM of AZD2461 in a time-dependent manner (3, 6, 12, and 24 h). ^*^p<0.05 compared to the untreated control groups

Treatment of PC-3 cells with AZD2461 significantly increased caspase3 activity at 0-12 h after treatment, in a time-dependent manner, reaching a maximum of four fold at 12 h after treated with 20 µM of AZD2461(*p <* 0.05; Fig. 5A). Likewise, AZD2461 significantly elevated caspase3 activity by 6 h in DU145 cells in a concentration-dependent manner, compared to that of controls (*p* < 0.05; Fig. 5B). However, this activity decreased at 24 h in both cell lines. Thus, caspase3 activity assay results are correlated with MTT and flow cytometric analysis in PC-3 and DU145 cell lines, indicating that apoptosis induction is dependent on the activation of caspase3.

**Analysis of** ***VEGF*** **mRNA levels**

**Fig. 6:**
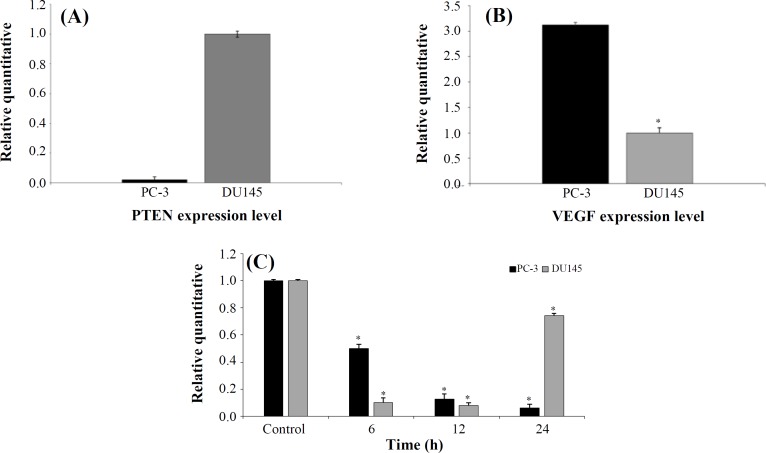
Real-time PCR analysis of *PTEN* and *VEGF* messenger RNA (mRNA) expression in DU145 and PC-3 cells. Relative (A) *PTEN*- and (B) -*VEGF* expression between two the cell lines.** (c)** Changes in *VEGF* mRNA levels following AZD2461 treatment (59.03 μM for DU145 and 36.48 μM for PC-3 cells according to IC_50_ values after 48 h of exposure) in a time-dependent manner (6, 12, and 24 h). Error bars represent SD. ^*^p < 0.05 compared to untreated control

Differential expressions of *PTEN* and* VEGF* in both PC-3 and DU145 cell lines were examined by real-time PCR using *SYBR* *Green* method. PC-3 cells was found to express significantly much lower levels of *PTEN*, while these cells displayed higher VEGF mRNA levels compared to DU145 cells (Fig. 6A and 6B). Evaluating the effect of AZD2461 on expression levels of the *VEGF* coding gene was conducted in a time-dependent fashion. The results demonstrated that *VEGF* expression level gradually reduced in the presence of PARP1 inhibitor over 6, 12, and 24 h after treatment compared to that of the untreated group (*p < *0.05; Fig. 6C). This result revealed that in all measured times, *VEGF* mRNA levels significantly decreased compared to the adjusted untreated cells (*p *< 0.05). Regarding DU145 cell line, mRNA expression level of *VEGF* reduced statistically at times 6 and 12 h (*p *< 0.05) although this value increased at 24 h (*p < *0.05). 

## DISCUSSION

The highly heterogeneous nature of Pca raises challenges and complexities toward the treatment process of high grade, advanced Pca patients^[^^33^^]^. Genomic-based classification provides the hope of discovering novel therapeutic molecular targets. PARP plays a pivotal function in the repair of SSBs and DSBs^[^^34^^]^. Subsequent to treatment with PARP inhibitors, PARP trapping results in the multiplication of the more DSBs. These DNA breaks would naturally be repaired by HRR pathway, otherwise cell death occurs^[^^35^^]^. Although PARP inhibitors have been found to be *synthetically lethal* in cells not expressing BRCA1/2, such mutations are not frequent in many tumors, including PCa malignant cells. Therefore, there is a reasonable concern towards discovering alternative determinants regarding sensitivity to PARP inhibitors^[^^36^^]^. A meta-analysis study in 2014 revealed that the therapeutic efficacy of olaparib in ovarian cancer is independent of BRCA1/2 mutation status^[^^3^^]^. Likewise, Mendes‐Pereira *et al.*^[^^37^^]^ announced that the clinical assessment of PARP inhibitors should be extended to possible BRCA-independent homologous-recombination deficient tumors, especially in *cells expressing very low levels of* PTEN, both in vitro and in vivo. 

*PTEN* has been found to be responsible for many nuclear functions, including transcriptional regulation of some HR-mediated genes, and more specifically, *RAD51* whose product is considered to be crucial for HR DNA DSBs repair^[^^38^^]^. McEllin and colleagues^[^^39^^]^ have previously reported that *PTEN* dysfunction in brain cells could be correlated with the enhanced sensitivity of these cells to PARP inhibitor, N-methyl-N’-nitro-N-nitrosoguanidine, since the DNA DSBs in astrocytes were not efficiently repaired. Additionally, PARP-1 inhibition disables the base excision repair leading to cell death^[^^40^^]^, and in HRR defective tumors such as *PTEN*- and *BRCA*-deficient or very low expressing backgrounds, employment of other error-prone DNA repair pathways can lead to severe genomic instabilities and decreased cell survival^[^^20^^]^. Also, the DNA repair-independent role of PARP1 inhibition in cell invasion has remained mainly unclear. Pharmacological inhibition of PARP1 significantly attenuated the metastatic potential of lung adenocarcinoma cells^[^^18^^]^. AZD2461 represents a useful inhibitor of PARP1 and has been found to potentiate the anti-proliferation effects of the DNA alkylating agents, not only in combination therapies but also as a monotherapeutic agent due to effective inhibition of DNA repair pathway. As a result, manipulating the DNA damage response might sensitize cells displaying lower levels of *PTEN* to PARP inhibitors more significantly compared to the cells with much higher baseline expression of this tumor suppressor. Although olaparib, as an orally active PARP inhibitor, has been reported to have the capability of inducing synthetic lethality in homozygous BRCA-deficient cells^[^^41^^]^, the efficacy and cell death-inducing effect of AZD2461, as a novel analogue of olaparib, have remained unclear, mostly due to efficacy problems that led *the phase II clinical trial* for this inhibitor to remain unfinished in 2011^[^^42^^]^. A study have also unraveled the differential PARP3 inhibitory activity of AZD2461 in HeLa KB31 and KBA1 cell lines^[^^20^^]^.

AZD2461 is considered as a poor substrate for multidrug resistance 1 (MDR1), and it can , therefore, be used for cell death-inducing in olaparib-resistant cancer cells overexpressing MDR1, which provides advantages over olaparib in monotherapy. Also, AZD2461 represents a potential preclinical tool in order to investigate the underlying mechanisms of PARPi resistance. Intriguingly, AZD2461 targets only proliferating cells with DSB repair dysfunction (i.e. *BRCA*- and *PTEN*-deficient or low expressed genetic backgrounds), supporting the mechanism regarding the conversion of SSBs into DSBs during DNA replication that eventually leads to cell death in tumor *cells *with* defective HR repair*. Our observations suggest that primary PC-3 cells harboring bi-allelic mutation in *PTEN*, showed increased sensitivity to PARP inhibition-mediated cell death. Our results regarding anti-proliferative effects of AZD2461 were *consistent with the findings of *Oplustil O'Connor et al.^[^^20^^]^*, revealing the advantages of AZD2461 monotherapy against BRCA1 mutant cancer cells. The current findings also* indicate that AZD2461 impedes the growth of PC-3 cells with higher capacity (lower IC_50_ values) compared to high *PTEN*-expressing DU145 cells, prompting the accumulation of intolerable levels of DNA damage in cycling cells. Annexin VPI double staining analysis established apoptosis as the primary cell death-inducing pathway in both cells. These results are in agreement with Weston *et al.*'s^[^^43^^]^ findings considering the growth inhibitory effect of olaparib in *ATM*-deficient lymphoid tumor cells, which have also impaired DNA repair capability. Although our data support the idea that PC-3 cells are significantly more responsive and also sensitive to PARP-specific inhibitors^[^^34^^]^, the possible underlying mechanisms causing the activation of other cell death pathways such as necrosis in *both cell lines have remained* unclear^[44]^. Moreover, we have evidenced that AZD2461 owns potential to enhance apoptotic-dependent cell death via increasing the activity of caspase3 significantly in PC-3 cells compared to DU145 cells. This result is in contrast to the previous study conducted by Yu *et al.*^[^^45^^]^ who declared that caspase-independent translocation of apoptosis-inducing factor from the mitochondria to the nucleus is impeded by N-methyl-N’-nitro-N-nitrosoguanidine, as a PARP-1 inhibitor. This observation suggests that caspase-PARP pathway might be responsible for apoptosis induction in PC-3 cells, but the complete understanding of the involved mechanism requires further investigations. Nevertheless, the response of the PC-3 cells to PARP inhibition is comparable to previous studies in BRCA1/2-defective breast carcinoma cells^[^^46^^,^^47^^]^.

Growth factors such as VEGF, EGF, and FGF2 have been found to overexpress in Pca cells^[^^24^^]^. VEGF and its receptors are known as the major regulators of tumor cell growth and metastasis over years. *VEGF* is expressed by various cancer cells, including most prostatic carcinoma cell lines. PC-3 cells have been proved to have higher metastatic potential compared to DU145 cells, which is likely due to higher baseline expression level of *VEGF* ^[^^25^^,^^48^^]^. In the present study, as expected, the relative expression of *VEGF* in DU145 cells was less than PC-3 cells, and treatment with IC_50_ concentrations of AZD2461 resulted in a significant decrease in *VEGF* mRNA levels of both PC-3 and DU145 compared to adjusted untreated cell lines in a time-dependent fashion. Altered expression levels of genes involved in angiogenesis are intriguing due to the reported vasoactivity of AG014699, a clinically active small molecule of PARPi^[^^49^^]^. Nevertheless, few studies have established the effects of PARP inhibition on angiogenesis, displaying both the inhibition and stimulation of angiogenesis^[^^50^^,^^51^^]^. Munoz-Gamez *et al.*^[^^52^^]^ have reported that the PARPi 4-amino-1,8-naphthalimide enhances the doxorubicin chemosensitivity in hepatocellular carcinoma cells by increasing apoptosis while reducing *EGFR* and *Bcl-xL* expressions. More studies are needed to clearly evaluate whether PARP inhibits or stimulates tumor cell progression pathways and assess simultaneously the vasoactive effect, but no earlier study was conducted regarding the investigation of the PARPi treatment of *VEGF* expression levels in Pca cells. These statements demonstrate that ADZ2461 is effective in reducing *VEGF* mRNA levels in both PC-3 and DU145 cells, treated with varying concentrations. However, dose- and time-dependent response varies in both cell lines. Our results provide a rationale for using AZD2461 as a novel, well-tolerated potent inhibitor of PARP1, which effectively inhibits DNA repair pathways and *yields* substantial advantages over olaparib as a *single-agent* therapy. Based on our findings, the anti-proliferative activity of AZD2461 was clearly observed in cells expressing significantly lower mRNA levels of *PTEN*. Thus, PARP inhibition can be regarded as a beneficial therapeutic strategy not only in BRCA1/2-deficient cells but also in Pca cells displaying low *PTEN* baseline expression due to their genetic backgrounds. Developing other specific inhibitors of PARP family gives the opportunity to maximize anti-tumor activity and therapeutic efficacy in these malignancies. Future studies are required on evaluating invasion or migration capacity of both cell lines when treated with AZD2461, using *in vitro* experiments such as Boyden Chamber and scratch assays. Also, either overexpressing *PTEN* in PC-3 cells or knocking down this gene in DU145 cells, which could have an impact on the susceptibility of Pca cells to AZD2461 should be studied in more details later.

Our data suggests that AZD2461 prohibits the proliferation of prostate tumor cells while raising the possibility that AZD2461 monotherapy could have direct anti-tumor effects, especially on the cancer cells harboring mutations in *PTEN *besides activating apoptosis-independent cell death pathways.
